# Editorial: Multi-scale dynamics modeling of brain physiological functions and pathological mechanisms

**DOI:** 10.3389/fnins.2023.1275481

**Published:** 2023-09-11

**Authors:** Jiajia Li, Rong Wang, Pan Lin, Miguel A. F. Sanjuan, Ying Wu

**Affiliations:** ^1^School of Information and Control Engineering, Xi'an University of Architecture and Technology, Xi'an, Shaanxi, China; ^2^School of Science, Xi'an University of Science and Technology, Xi'an, China; ^3^Center for Mind & Brain Sciences, Hunan Normal University, Hunan, Changsha, China; ^4^Nonlinear Dynamics, Chaos and Complex Systems Group, Departamento de Física, Universidad Rey Juan Carlos, Madrid, Spain; ^5^State Key Laboratory for Strength and Vibration of Mechanical Structures, School of Aerospace Engineering, Xi'an Jiaotong University, Xi'an, Shaanxi, China

**Keywords:** dynamics modeling, neuron network, physiological functions, pathological mechanisms, brain function

## 1. Introduction

The human brain is an extraordinary organ, sometimes referred to as the most complex system since it is responsible for our thoughts, emotions, and actions. Understanding how the brain works and unraveling the mechanisms underlying neurological disorders are long-outstanding challenges in neuroscience. In this editorial article, we delve into the exciting realm of multi-scale dynamics modeling of brain physiological functions and pathological mechanisms. This Research Topic in Frontiers in Neuroscience brings together a collection of groundbreaking studies that shed light on the complex dynamics of the brain, offering new insights into both normal brain function and the underlying causes of neurological disorders.

## 2. Exploring the multi-scale dynamics

As is well known, the brain operates across multiple scales, from the interactions between individual neurons to the coordination of large-scale networks. Traditional reductionist approaches have provided valuable insights into specific aspects of the brain function. However, a comprehensive and holistic understanding of the brain dynamics necessitates a multi-scale perspective that integrates information across different levels of organization such as the neuron scale, the neuron network scale, and the brain network scale. Cutting-edge research employing innovative modeling techniques to capture the complexity of brain dynamics across various scales (Xu and Kang; Liu and Sun) is showcased here.

## 3. Unraveling brain physiological functions

Several articles focus their attention on unraveling the fundamental physiological functions of the brain. By combining experimental data with computational models, researchers have made significant strides in elucidating the mechanisms underlying sensory perception (Xu and Kang), memory formation (Liu and Sun), and attention (Wang et al.). These studies provide valuable insights into the dynamic interplay between different brain regions and shed light on the neural mechanisms that give rise to our cognitive abilities.

## 4. Unveiling pathological mechanisms

Neurological disorders pose significant challenges to human health and wellbeing. From this perspective, several articles delve into the pathological mechanisms underlying conditions such as epilepsy (Fan et al.; Jiang et al.), Alzheimer's disease (Li et al.), acoustic neuroma (Zhang et al.), pituitary adenoma (Wang et al.), and sleep disorders (Li and Dong). By employing multi-scale modeling approaches, researchers have made remarkable progress in unraveling the complex interactions between genetic, cellular, and network-level abnormalities that contribute to these disorders. Most importantly, this deeper understanding opens new avenues for the development of targeted therapeutic interventions.

## 5. Bridging the gap between theory and clinical applications

The studies presented here not only advance our theoretical understanding of brain dynamics, but also have important implications for clinical applications (Wang et al.; Xu et al.; Zhang et al.). By elucidating the mechanisms underlying neurological disorders, these findings pave the way for the development of novel diagnostic tools, personalized treatment strategies, and therapeutic interventions. The integration of multi-scale modeling with clinical data holds great promise for improving patient outcomes and transforming the field of neurology.

## 6. Conclusions

The multi-scale dynamics modeling of brain physiological functions and pathological mechanisms represents a paradigm shift in neuroscience research. This Research Topic in Frontiers in Neuroscience brings together a diverse range of studies that push the boundaries of our understanding of the brain. By integrating knowledge from different scales, these studies provide a comprehensive picture of brain dynamics and offer new avenues for tackling neurological disorders. The insights gained from this research have the potential to transform our approach to brain health and pave the way for more effective treatments in the future.

## Author contributions

YW: Writing—review and editing, Supervision. JL: Writing—original draft. RW: Writing—review and editing. PL: Writing—review and editing. MS: Writing—review and editing.

